# COVID-19 prognosis: what we know of the significance and prognostic value of liver-related laboratory parameters in SARS-CoV-2 infection 

**Published:** 2020

**Authors:** Davood Bashash, Meysam Olfatifar, Farzad Hadaegh, Hamid Asadzadeh Aghdaei, Mohammad Reza Zali

**Affiliations:** 1 *Proteomics Research Center, Department of Hematology and Blood Banking, School of Allied Medical Sciences, Shahid Beheshti University of Medical Sciences, Tehran, Iran*; 2 *Gastroenterology and Liver Diseases Research Center, Research Institute for Gastroenterology and Liver Diseases, Shahid Beheshti University of Medical Sciences, Tehran, Iran*; 3 *Prevention of Metabolic Disorders Research Center, Research Institute for Endocrine Sciences, Shahid Beheshti University of Medical Sciences, Tehran, Iran*; 4 *Basic and Molecular Epidemiology of Gastrointestinal Disorders Research Center, Research Institute for Gastroenterology and Liver Diseases, Shahid Beheshti University of Medical Sciences, Tehran, Iran*

**Keywords:** COVID-19, Prognosis, Liver, Albumin, Bilirubin, Aminotransferase

## Abstract

**Aim:**

The present study aims to evaluate the prognostic value of liver-related laboratory parameters in COVID-19.

**Background:**

This is not the first nor will it be the last time that a member of the β-coronaviruses wages a full-scale war against human health. Notwithstanding atypical pneumonia being the primary symptom, the emergence of severe disease mainly resulting from the injury of non-pulmonary organs leaves no alternative, in some cases, other than a dreadful death.

**Methods:**

To provide a well-conceptualized viewpoint representing the prognostic values of liver-related laboratory parameters in COVID-19, a meta-analysis was performed with the calculation of mean difference and 95% conﬁdence intervals of alanine aminotransferase (ALT), aspartate aminotransferase (AST), total bilirubin (Bili), and albumin (Alb) in severe and non-severe COVID-19 patients.

**Results:**

While severe COVID-19 cases displayed higher values of ALT, AST, and Bili compared to non-severe patients (mean differences of 7.48, 12.07, and 3.07, respectively), the value of Alb was significantly lower in severe cases (mean differences of -6.15). There was also a correlation between alterations in all of the parameters; however, only correlations between ALT and Bili (R=0.98, p=0.0031), and Bili and Alb (R=-1, p=0.0012) were significant.

**Conclusion:**

Abnormal values of liver-related examinations outwardly contribute to reflect the progression of the disease toward an unfavorable outcome. Therefore, careful scrutiny of these parameters will provide clinicians with invaluable information regarding SARS-CoV-2 infection, at least in terms of liver injury.

## Introduction

 An outbreak of pneumonia of unknown etiology in Wuhan City, China, prompted the World Health Organization (WHO) to issue a public health warning emergency in early 2020 (1). This is not the first nor will it be the last time that a member of the β-coronaviruses (CoVs), as enveloped positive-strand RNA pathogens (2), threatens human health. While the severe acute respiratory syndrome (SARS)-CoV and the Middle East respiratory syndromes (MERS)-CoV have caused about 10,000 cumulative cases with mortality rates of 10% and 35%, respectively (3), the situation in this recent case is quite eerie considering that the novel identified CoV (designated as SARS-CoV-2 by the WHO in February, 2020) has potentially more secretive characteristics to be discovered.

The spread of SARS-CoV-2 has already met and surpassed the necessary criteria for the announcement of a pandemic (4), affecting more than 2,500,000 people in 210 countries in fewer than 4 months (https://www.who.int/). Unfortunately, not only the total number of infected patients is growing unceasingly, but also the dreadful statistics of deaths are following an exponential trend in most countries of the world. Despite atypical pneumonia being the primary symptom (5), there may be no end, in some cases, other than death due to the emergence of severe disease resulting from the injury of several non-pulmonary organs, including the liver (6). Notwithstanding that Wynants et al. (7) introduced age, sex, features derived from computed tomography scans, C reactive protein, lactic dehydrogenase, and lymphocyte count as the most reported predictors of severe prognosis in patients with COVID-19, there are several lines of evidence unveiling non-disclosed mysteries relevant to disease prognosis. In this vein and taking advantage of the results of several recent studies reporting altered values of liver enzymes in patients with COVID-19 (8), it might not be unrealistic to assume that abnormal levels of these factors may potentially serve as simple and readily available biomarkers to predict disease severity. Although the rapidly evolving nature of COVID-19 together with some limitations such as low sample size, poor description of the analytical performance characteristics of the methods used, different measurement units, and variable panels applied for patients are among major concerns of the present study, we hope that the results of our meta-analysis will help clinicians manage the disease as effectively as expected. 

## Methods

In order to provide a well-conceptualized viewpoint expressing the prognostic value of liver-related laboratory parameters in COVID-19, the national library of medicine Medline/Pubmed was searched using the keywords “laboratory” OR “ALT” OR “AST” OR “Liver” OR “alanine aminotransferase” OR “aspartate aminotransferase” AND “COVID-19” OR “coronavirus 2019” OR “2019-nCoV” OR “SARS-CoV-2” for articles published between December 2019 and the present time (i.e., April 15, 2020), with no restrictions. The results of the initial search strategy were first screened by title and abstract, and then the full texts of relevant articles representing information on the indicated parameters in COVID-19 patients with a clinically validated deﬁnition of severe disease were ﬁnally selected. To strengthen the analysis, the reference lists of relevant documents were also scrutinized. Next, a meta-analysis was performed with the calculation of mean difference (MD) and 95% conﬁdence intervals (95% CIs) of ALT, AST, Bili, and Alb in severe and non-severe patients. To do so, the standard deviations (SDs) of selected studies were estimated based on means and their related CIs. The statistical analysis was implemented in the R “meta” package (9). Subgroup analysis was also applied by the study deﬁnition of severity. Heterogeneity between studies was estimated using the I2 method, where I2 values of 25%, 50%, and 75% were defined as low, moderate, and high heterogeneity, respectively. In addition, to assess whether there is a possible correlation between alteration values of ALT, AST, Bili, and Alb, the Pearson correlation coefficient analysis was applied using the ggplot2 package in R. A probability value of less than 0.05 was considered statistically significant. 

## Results

Overall, 1912 articles were identified using the indicated criteria in the initial search and by inspecting the reference lists. Among these articles, there were 285 letters, 189 reviews, 141 editorials, 61 case reports, 53 comments, 6 guidelines, and 1 book. Not only were these items excluded, but also excluded were those articles that did not give information on the indicated parameters and those that provided incomplete information. Out of 17 selected articles, 12 studies reported alteration rates (%) of liver-related biochemical parameters in COVID-19 (Table 1). Additionally, 10 articles (in which 5 were shared) reported values of ALT, AST, Bili, and Alb in both severe and non-severe COVID-19 cases. 

**Table 1:** Alteration rate (%) of liver-related biochemical parameters in COVID-19

	Yang et al. (24)	Chen et al. (25)	Liu et al. (26)	Chen et al. (27)	Cao et al. (20)	Guan et al. (10)	Chen et al. (28)	Chen et al. (29)	Zhou et al. (30)	Wan et al. (21)	Huang et al. (22)	Xu et al. (31)
No. of cases (Severe)	149 (0)	99 (17)	12 (6)	29 (14)	198 (19)	1099 (173)	175 (N/R)	9 pregnant	191 (54)	135 (40)	41 (13)	62 (1)
Age (year)	45	56	54	56	50	47	46	30	56	40	49	41
Female (%)	45.6%	32%	33%	28%	49%	41.9%	53%	100%	38%	46.7%	27%	44%
ALT	↑12%	↑28%	↑17%	↑17%	↑10.8%	↑21.3%	↑19%	↑33%	↑31%			
AST	↑18%	↑35%	↑8%	↑24%	↑17.4%	↑22.2%	↑16%	↑33%		↑22%	↑37%	↑16%
Bilirubin	↑2.7%	↑18%	↑0%	↑3%	↑2.6%	↑10.5%						
Albumin	↓6%	↓98%	↓50%	↓52%	↓40%							

**Figure 1:**
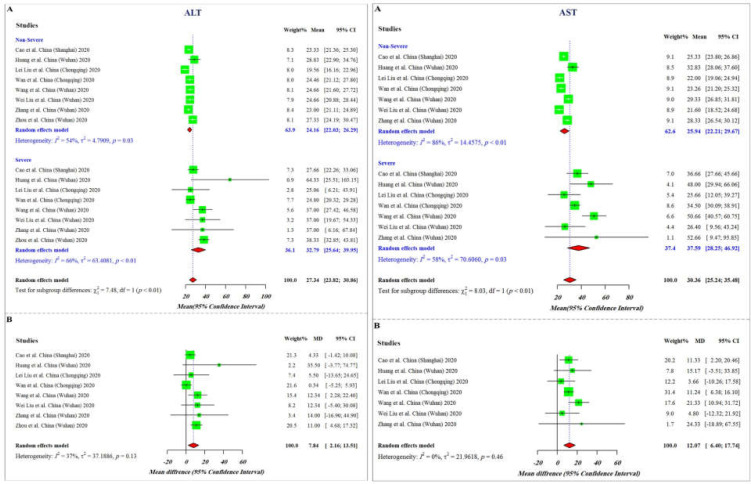
Forest plot of **A)** mean and **B)** mean difference in alanine aminotransferase (ALT) and aspartate aminotransferase (AST) values between severe and non-severe COVID-19 patients

The main features and severity definition of these studies (totaling 2302 patients, 476 of whom (20.06%) had severe disease) are summarized in Table 2. Since the results presented by Guan et al. (10) were not in a synchronized manner with other studies, this study was not included in the current meta-analysis. 

**Figure 2:**
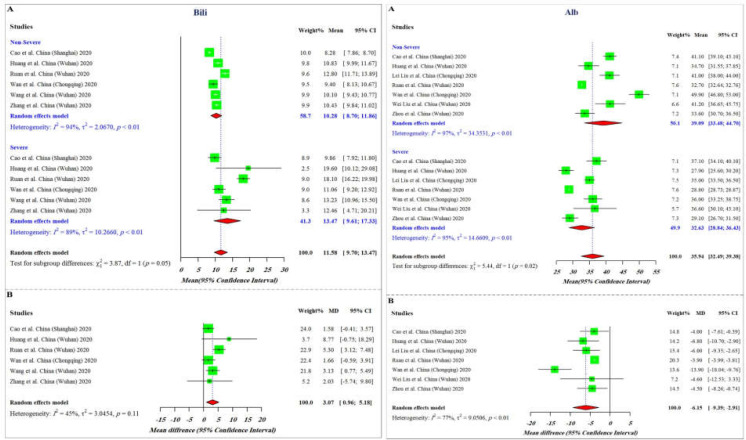
Forest plot of **A)** mean and **B)** mean difference in total bilirubin (Bili) and albumin (Alb) values between severe and non-severe COVID-19 patients

**Figure 3:**
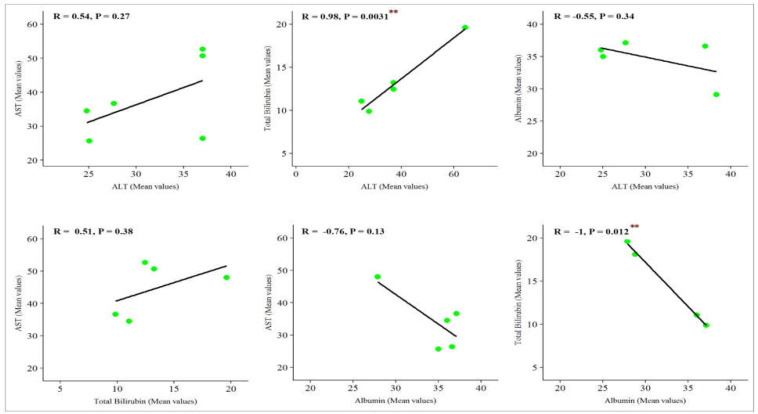
Correlation analysis between liver-related laboratory parameters. Although there is correlation between alterations of all these parameters, the resulting data show that a significant correlation exists between alteration of ALT and Bili, and Bili and Alb

The results of the current meta-analysis revealed that severe COVID-19 cases displayed higher values of ALT, AST, and Bili compared to patients with non-severe disease (mean differences of 7.48, 12.07, 3.07, respectively), while the value of Alb was significantly lower in severe cases (mean differences of -6.15) (Figures 1 and 2). As represented, among all these parameters, the difference range of AST is more meaningful between non-severe and severe cases, proposing it as a more reliable factor for predicting COVID-19 severity. Analysis of the pooled results of the selected studies further confirmed the findings of the subgroup analysis. Overall and taking advantage of our data showing that the mean values of ALT, AST, and Bili in severe patients are significantly higher than those of non-severe patients (X2=7.48, p<0.01; X2=8.03, p<0.01; X2=3.87, p<0.05, respectively) together with a decreased level of Alb in severe cases (X2=5.44, p<0.02), it is reasonable to propose that alterations in liver-related biochemical parameters may effectively contribute to reflect the progression of disease toward an unfavorable clinical picture. Having established that there was a significant difference between the values of the indicated parameters among severe and non-severe patients, statistical correlation analysis was then used to evaluate whether there are significant correlations between the alteration values of these factors in the severe group. Although there was a correlation between alterations of all these parameters, the resulting data showed that a significant correlation exists only between alterations of ALT and Bili (R=0.98, p=0.0031), and Bili and Alb (R=-1, p=0.0012) (Figure 3).

**Table 2:** Main features and values of liver-related biochemical parameters in severe and non-severe COVID-19 patients

	Wan et al. (21)	Huang et al. (22)	Cao et al. (20)	Ruan et al. (32)	Zhang et al. (33)	Wang et al. (34)	Wei et al. (35)	Lei Liu et al. (36)	Zhou et al. (30)	Guan et al. (10)
Features										
No. of cases (Severe)	135 (40)	41 (13)	198 (19)	150 (68)	221 (55)	138 (36)	78 (11)	51 (7)	191 (54)	1099 (173)
Age (Years)	40	49	50	58	55	56	38	45	56	47
Female (%)	46.7%	27%	49%	32%	51%	45.7%	50%	37.3%	38%	41.9%
Country (City)	China (Chongqing)	China (Wuhan)	China (Shanghai)	China (Wuhan)	China (Wuhan)	China (Wuhan)	China (Wuhan)	China (Chongqing)	China (Wuhan)	China (30 provinces)
Year	2020	2020	2020	2020	2020	2020	2020	2020	2020	2020
Severity definition	ICU admission Mech. ventilation	ICU admission	ICU admission	Death	WHO guidelines (37)	ICU admission	ICU admission Mech. ventilation Death	WHO guidelines	Death	ICU admission Mech. ventilation Death
ALT										
Total	26 (12.9-33.15)	32 (21-50)	23 (15-33)		23 (16-39)	26 (16-40)	18.1 (13.7, 30.7)	18 (14-30)	30 (17–46)	↑41%
Non-severe	21.7 (14.8-36.9)	27 (19.5-40)	22 (15-33)	48.68 (83.1)	22 (14-33)	23 (15-36)	18.5 (12.5, 27.7)	18 (14-32)	27 (15–40)	↑19.8%
Severe	26.6 (14.5-33.3)	49 (29-115)	30 (19-34)	170.8 (991.6)	32 (22-57)	35 (19-57)	17.4 (13.9, 43.9)	26 (10-30)	40 (24–51)	↑28.1%
AST										
Total	33.4 (27.8-43.7)	34 (26-48)	26 (20-34)		29 (22-49)	31 (24-51)	20.5 (13.8, 33.5)	21 (16-30)		↑22.2%
Non-severe	22.4 (16.9-30.5)	34 (24-40.5)	24 (19-33)	40.7 (57.8)	27 (20-38)	29 (21-38)	20 (13.9, 30.9)	21 (16-29)		↑18.2%
Severe	33.6 (25.7-44.2)	44 (30-70)	33 (26-51)	288.9 (1875.5)	51 (29-78)	52 (30-70)	21.6 (12, 45.6)	29 (14-34)		↑39.4%
Total Bilirubin										
Total	8.6 (5.9-13.7)	11.7 (9.5-13.9)	8.1 (6.5-10.6)		10 (8-14.2)	9.4 (8.4-14.1)				↑10.5%
Non-severe	8.6 (5.6-14)	10.8 (9.4-12.3)	8.0 (6.5-10.35)	12.8 (6.8)	9.6 (7.9-13.8)	9.3 (8.2-12.8)				↑9.9%
Severe	9.8 (7.8-15.6)	14 (11.9-32.9)	9.0 (7.6-13.0)	18.1 (10.7)	11.4 (8.6-17.4)	11.5 (9.6-18.6)				↑13.3%
Albumin										
Total	40.5 (37-43.4)	31.4 (28.9-36)	40.92 (38-43.1)				40.47 (35.26-45.68)	40 (36-43)	32·3 (29·1–35·8)	
Non-severe	49.9 (37.4-43.6)	34.7 (30.2-36.5)	41.1 (39-43)	32.7 (3.8)			41.2 (36.7-45.8)	41 (38-44)	33·6 (30·6–36·4)	
Severe	36 (33-38.5)	27.9 (26.3-30.9)	37.1 (33-39)	28.8 (3.8)			36.6 (30.2-43.2)	35 (33-36)	29·1 (26·5–31·3)	

Mech. Ventilation: Mechanical ventilation; ICU: Intensive care unit.

While several limitations, such as low sample size, poor description of disease outcome, and sampling time together with the adoption of different measurement units and methods, adversely affected this analysis, it is hoped that the results of the present study will shed more light on the values of these parameters in COVID-19 patients.

## Discussion

While SARS-CoV-2 infection mainly causes mild pulmonary symptoms, the situation of patients with this infection may get much worse and even lead to death due to multi-organ failure. Among the injury of non-pulmonary organs, liver impairment, as also reported by up to 60% in SARS (11), has recently been the focus of many studies in COVID-19. Although for the time being, the reason and precise mechanisms behind the hepatic injury are still unclear, several hypotheses have been put forward. The ability of SARS-CoV to bind to the angiotensin-converting enzyme 2 (ACE2) receptor (12) together with the enriched expression of this receptor in cholangiocytes (13) ignites the assumption that SARS-CoV-2 might directly bind to ACE2-positive cholangiocytes to dysregulate liver function. In addition, it may not be unrealistic to propose that contagion from this deadly virus may aggravate cholestasis in patients with primary biliary cholangitis. Direct damage to liver cells caused by viral infection is another recommended mechanism. In a study by Yeo C. et al. published by The Lancet Gastroenterology & Hepatology (14), SARS-CoV-2 RNA has been detected in the stool and blood samples of COVID-19 patients, implying the possibility of viral exposure in the liver. Immune-mediated inflammation, such as cytokine storm syndrome (CCS) and pneumonia-associated hypoxia (15) along with drug-induced hepatotoxicity (16), are also among mechanisms underlying hepatic injury in COVID-19. Notably, the latter may explain, at least partly, the great discrepancy reported across different cohorts. Considering the results of the first four autopsies from New Orleans which proposed thrombotic microangiopathy within the alveolar capillaries as a fatal mechanism in severe COVID-19 patients (17), it may be concluded that damage to the small blood vessels of vital tissues, eventually followed by intravascular coagulopathy, will probably be responsible for multi-organ failure. By and large, at the time of writing of this article, there has been a lack of research regarding the intricate mechanism beneath this issue; this lack necessitates the planning of further tudies to clarify the precise association between COVID-19 and liver injury.

The results of a recent study revealed that 2–11% of patients with COVID-19 had liver comorbidities, and 14–53% cases reported abnormal levels of ALT and AST (6). It is worth noting that the issue does not end here, because the increased values of these parameters were also reported to be closely related to the severity of the disease (18). In a study in The Lancet Infectious Disease, Shi h. et al. (19) reported that asymptomatic COVID-19 patients had significantly lower mean concentrations of AST (30.2 U/L, p=0.0026) than did patients in symptomatic groups, which is in agreement with the current results, proposing that alteration of AST values with a mean difference value of 12.07 between severe and non-severe cases is probably more trustworthy than other parameters. The elevations in ALT and AST were also reported in 15.8% and 42.1% of 19 severe patients entailing specialized management in intensive care units (ICU) compared with 10.2% and 14.8% of 179 patients who did not require care in the ICU, respectively. In addition, the researchers reported alteration rates of 5.2% vs. 2.3% and 84.2% vs. 35.8% in total bilirubin and albumin in severe vs. non-severe cases, respectively (20). Increased values of ALT, AST, and total bilirubin together with a decreased level of albumin were also reported in two other studies (21, 22). Consistently, the results of the current meta-analysis revealed that altered values of liver-related biochemical factors in severe patients are significantly greater as compared with non-severe groups, proposing these factors as valuable laboratory criteria for predicting COVID-19 prognosis, at least in terms of liver injury. 

In agreement with the current findings, a recent meta-analysis also reported that abnormal values of the indicated parameters could mirror a picture of liver impairment in patients who develop the severe form of the disease (23). Pearson’s correlation analysis also revealed that there was a correlation between alterations in all of these parameters; however, a significant correlation exists only between the alterations in ALT and Bili, and Bili and Alb. By and large, the present study highlights that careful scrutiny of liver-related laboratory examinations retains a specific clinical significance in this infection, and abnormality in these parameters seemingly contributes to reflect the progression of COVID-19 toward an unfavorable outcome; however, future research shall be planned to provide new clues and data both to confirm the current data and to identify other biomarkers of poor outcomes in this disease.
